# Consuming the daily recommended amounts of dairy products would reduce the prevalence of inadequate micronutrient intakes in the United States: diet modeling study based on NHANES 2007–2010

**DOI:** 10.1186/s12937-015-0057-5

**Published:** 2015-09-04

**Authors:** Erin E Quann, Victor L Fulgoni, Nancy Auestad

**Affiliations:** 1 Dairy Management Inc., 10255 West Higgins Road, Suite 900, 60018 Rosemont, IL USA; 2 Nutrition Impact, LLC, 9725 D Drive North, 49014 Battle Creek, MI USA; 3 Nutrition Insights, LLC, 44 S 2740 West, 84770 St, George, UT USA

**Keywords:** Nutrients, Nutrient adequacy, Dairy, Nutrition and health, NHANES

## Abstract

**Background:**

A large portion of Americans are not meeting the Dietary Reference Intakes (DRI) for several essential vitamins and minerals due to poor dietary choices. Dairy products are a key source of many of the nutrients that are under consumed, but children and adults do not consume the recommended amounts from this food group. This study modeled the impact of meeting daily recommended amounts of dairy products on population-based nutrient intakes.

**Methods:**

Two-day 24-h dietary recalls collected from participants ≥2 years (*n* = 8944) from the 2007–2010 What We Eat in America, National Health and Nutrition Examination Survey (NHANES) were analyzed. Databases available from the WWEIA/NHANES and the United States Department of Agriculture (USDA) were used to determine nutrient, food group, and dietary supplement intakes. Modeling was performed by adding the necessary number of dairy servings, using the dairy composite designed by USDA, to each participant’s diet to meet the dairy recommendations outlined by the 2010 Dietary Guidelines for Americans. All analyses included sample weights to account for the NHANES survey design.

**Results:**

The majority of children 4 years and older (67.4–88.8 %) and nearly all adults (99.0–99.6 %) fall below the recommended 2.5-3 daily servings of dairy products. Increasing dairy consumption to recommended amounts would result in a significant reduction in the percent of adults with calcium, magnesium, and vitamin A intakes below the Estimated Average Requirement (EAR) when considering food intake alone (0–2.0 vs. 9.9–91.1 %; 17.3–75.0 vs. 44.7–88.5 %; 0.1–15.1 vs. 15.3–48.0 %, respectively), as well as food and dietary supplement intake. Minimal, but significant, improvements were observed for the percent of people below the EAR for vitamin D (91.7–99.9 vs. 91.8–99.9 %), and little change was achieved for the large percentage of people below the Adequate Intake for potassium.

**Conclusions:**

Increasing dairy food consumption to recommended amounts is one practical dietary change that could significantly improve the population’s adequacy for certain vitamins and minerals that are currently under-consumed, as well as have a positive impact on health.

## Background

Recommendations in the 2010 Dietary Guidelines for Americans (DGA) vary widely in scope and intended impact. Some recommendations are intended to have a focused scope such as reduced consumption of trans-fats to promote cardiovascular health. Other recommendations promote a wide range of health benefits such as consuming the recommended servings from each food group to ensure nutrient adequacy, reduce chronic disease risk, and help build an overall healthy diet pattern that meets nutrient needs within calorie goals [1]. Likewise, the recommendation to consume more servings of low-fat and fat-free dairy foods is intended to lead to multiple nutrition and health benefits. These include increased consumption of calcium, vitamin D, potassium and other nutrients often under-consumed, along with health benefits such as improved bone health, and reduced risk of cardiovascular disease and type II diabetes.

Dairy products make a significant contribution to the U.S. population’s nutrient intakes, including nearly 60 % of the vitamin D, half of the calcium and about 15–25 % of the potassium, protein, vitamin A, vitamin B12, phosphorus, zinc and riboflavin intakes [2]. In fact, milk is the leading food source of three of the four nutrients of public health concern (calcium, vitamin D and potassium) for children 2–18 years old and adults 19 years and older [3, 4]. This nutritional contribution represents a fraction of the potential nutritional impact of dairy foods because dairy foods are under-consumed. In 2001–2004, only 15 % of Americans ages 2 years and older consumed the recommended servings of dairy products (milk, cheese, yogurt) per day with less dairy consumed by older adults compared to children [5]. The 2010 DGA recommends Americans over the age of 9 years consume 3 servings daily; however, the more recent National Health and Nutrition Examination Survey (NHANES) 2009–2010 indicates that Americans ages 2 and older consume only 1.9 servings of dairy products per day on average [6]. Dairy consumption continues to decline with increasing age, starting early in childhood [7]. Given the importance of dairy’s nutrients during growth and development, and the link to improved health outcomes for adults, it is important to determine the most effective dietary strategies that help to reverse these poor dairy consumption trends.

Using dietary modeling (NHANES 1999–2004), Nicklas, et al. [8] found that the prevalence of inadequate calcium, potassium and magnesium intakes in different age groups could be reduced if additional servings of dairy foods were consumed. It is timely to reevaluate this finding in light of more recent data, updated calcium and vitamin D DRI’s by the Institute of Medicine’s Food and Nutrition Board (including an estimated average requirement (EAR) rather than the previous adequate intake (AI) for calcium and vitamin D) [9, 10], and the 2010 DGA increased recommended dairy servings from 2 to 2.5 for 4–8 year olds [1].

This study uses the NHANES 2007–2010 surveys to model the impact of Americans meeting the daily recommended amounts of dairy products on population-based vitamin and mineral intakes. Although the 2010 DGA recommends obtaining adequate intake of nutrients from foods alone [1], dietary supplements also make a significant contribution to some vitamin and mineral intakes in the U.S. [11]. This study, therefore, also assessed the impact that additional dairy products would have on nutrient adequacy when considering both food and dietary supplement use. Our hypothesis is that consuming the daily recommended number of dairy servings will significantly improve the diets of Americans by decreasing the proportion of children and adults with inadequate micronutrient intakes, in particular in population subgroups that consume the least amount of dairy and have the largest nutrient shortfalls, such as teen girls and older adults.

## Methods

### Study population

Participants were children 2 years of age and older (*n* = 8944) from the NHANES 2007–2010. The NHANES is now conducted on a continual basis by the National Center for Health Statistics of the Centers for Disease Control and Prevention and details regarding the survey design, content, operations and procedures are available online [12].

### Intake measures

Dietary data were obtained from two 24-h dietary recalls administered using an automated multiple-pass method [13]. The first was obtained at the original interview (Day 1) and the second (Day 2) was obtained several days later via telephone. Parents/guardians provided the 24-h dietary recalls of children 2–5 years; children 6–11 years were assisted by an adult; all others provided their own recalls. Only recall data deemed complete and reliable by the United States Department of Agriculture (USDA) Food Surveys Research Group were included in the analyses (excluded *n* = 129). Pregnant or lactating females (*n* = 153) and children reporting consumption of breast milk (*n* = 5) were excluded from the sample. Detailed descriptions of the dietary interview methods are provided in the NHANES Mobile Examination Center In-Person Dietary Interviewers Procedures Manual, which includes pictures of the Computer-Assisted Dietary Interview system screens, measurement guides, and charts used to collect dietary information [14]. The NHANES has stringent protocols and procedures that ensure confidentiality and protect individual participants from identification using federal laws [15]. This study was a secondary data analysis which lacked personal identifiers; therefore, this study did not require approval of an Institutional Review Board.

The USDA Food and Nutrient Database for Dietary Studies v. 4.1 [16] was used to determine the nutrient content of foods in 2007–2008 NHANES survey foods, and the Food and Nutrient Database for Dietary Studies v. 5.0 [17] was used to determine the nutrient content of foods contained in 2009–2010 NHANES survey foods. Servings, in cup equivalents, of total dairy, milk, cheese, and yogurt were determined using Food Patterns Equivalent Database for NHANES 2007–2008 [18] and NHANES 2009–2010 [19].

Modeling was performed by adding the necessary number of dairy servings to each participant’s diet to meet DGA recommendations. The nutrient composite of the dairy servings added to each participant’s diet was that used by USDA to develop recommended food patterns to meet DGA recommendations [20]. According to USDA, one cup equivalent of dairy contains 81 kcal, 8.5 g protein, 0.87 g saturated fat, 298 mg calcium, 20 mg magnesium, 99 Retinol Activity Equivalents (RAE) vitamin A, 1.55 μg vitamin D, 237 mg potassium, and 181 mg sodium.

### Statistical analyses

The National Cancer Institute method [21, 22] was used to estimate usual intake of total dairy and of selected nutrients currently being consumed to assess current intake, the percentage of the population meeting DGA dairy recommendations, and nutrient adequacy. Usual intake analyses were repeated after modeling inclusion of additional dairy servings to meet DGA recommendations. The two days of intake, using first day sampling weights, were used to obtain necessary variance estimates. The National Cancer Institute SAS (SAS Institute, Inc., Cary, NC) macros Mixtran v.1.1 and Distrib v.1.1 were used to generate parameter estimates after covariate adjustment and to estimate the distribution of usual intake via the Monte Carlo method, respectively. Covariates for these analyses were sequence of participant’s intake (Day 1 or Day 2) and a variable for weekday/weekend consumption. The percent of the population below the DGA dairy recommendations, below the EAR for protein, calcium, magnesium, vitamin A, and vitamin D and above the AI for potassium were also examined as recommended by the DRI applications publication [23]. For all analyses study-specific dietary four-year sample weights were used to adjust the variance for the complex sample design of NHANES [24] using the statistical package SAS (version 9.2; SAS Institute, Cary, NC). The population was separated into the following age groups: 2–3, 4–8, 9–18, 19–50, 51–70, 71+ years of age for gender combined and for males and female separately. Meaningful differences in percentage of the population with usual intakes below the EAR or above the AI, were determined by using non-overlapping 95^th^ percentile confidence limits between current intakes and modeled intakes.

## Results

### Dairy intake

Daily consumption of milk, cheese and yogurt by different age and gender groups is shown in Table 1. Milk consumption is highest among 2–3 and 4–8 year olds who consume 1.9 and 1.5 servings of milk a day, respectively for both genders combined, compared to less than one serving a day for adults. Women consume less milk than men. On the other hand, cheese consumption is low among 2–3 year olds, increases to 0.6–0.8 servings a day in adulthood, and drops off again at ages 71+ to only one–third of a serving each day. Yogurt consumption is not a major contributor to the number of dairy servings in any population group.

**Table 1:** Intake of total dairy, milk, cheese and yogurt and percentage meeting total dairy recommendations in America

		Milk	Cheese	Yogurt	Total dairy	% Below dairy recommendation
	n	Cup equivalents/day				
All						
2–3 years	477	1.8 ± 0.1	0.4 ± 0.1	0.1 ± 0.0	2.4 ± 0.1	16.2 ± 1.7
4–8 years	958	1.5 ± 0.1	0.6 ± 0.0	0.1 ± 0.0	2.1 ± 0.1	67.4 ± 1.5
9–18 years	1689	1.2 ± 0.1	0.8 ± 0.0	0.0 ± 0.0	2.1 ± 0.1	88.8 ± 0.9
19–50 years	3123	0.8 ± 0.0	0.8 ± 0.0	0.1 ± 0.0	1.7 ± 0.1	98.0 ± 0.3
51–70 years	1771	0.8 ± 0.0	0.6 ± 0.0	0.1 ± 0.0	1.5 ± 0.1	99.0 ± 0.3
71+ years	926	0.9 ± 0.0	0.3 ± 0.0	0.1 ± 0.0	1.3 ± 0.0	99.6 ± 0.2
Females						
2–3 years	221	2.0 ± 0.1	0.4 ± 0.1	0.1 ± 0.0	2.5 ± 0.2	18.6 ± 1.9
4–8 years	459	1.4 ± 0.1	0.6 ± 0.1	0.1 ± 0.0	2.1 ± 0.1	77.3 ± 2.5
9–18 years	823	1.0 ± 0.1	0.7 ± 0.0	0.0 ± 0.0	1.7 ± 0.1	99.2 ± 0.3
19–50 years	1593	0.8 ± 0.0	0.6 ± 0.0	0.1 ± 0.0	1.4 ± 0.0	99.6 ± 0.1
51–70 years	867	0.7 ± 0.0	0.5 ± 0.0	0.1 ± 0.0	1.3 ± 0.0	99.9 ± 0.1
71+ years	480	0.8 ± 0.0	0.4 ± 0.0	0.1 ± 0.0	1.3 ± 0.0	100.0 ± 0.0
Males						
2–3 years	256	1.7 ± 0.1	0.4 ± 0.1	0.1 ± 0.0	2.3 ± 0.1	14.0 ± 2.3
4–8 years	499	1.5 ± 0.0	0.5 ± 0.0	0.1 ± 0.0	2.1 ± 0.1	58.3 ± 1.9
9–18 years	866	1.5 ± 0.1	0.9 ± 0.1	0.0 ± 0.0	2.4 ± 0.2	77.9 ± 1.8
19–50 years	1530	0.9 ± 0.1	1.0 ± 0.1	0.0 ± 0.0	1.9 ± 0.1	96.3 ± 0.6
51–70 years	904	1.0 ± 0.1	0.7 ± 0.1	0.0 ± 0.0	1.8 ± 0.1	98.0 ± 0.5
71+ years	446	1.0 ± 0.1	0.3 ± 0.0	0.0 ± 0.0	1.3 ± 0.1	99.0 ± 0.4

With the exception of ages 2–3 years, the majority of children do not consume the recommended amounts of dairy products. In both the 4–8 and 9–18 year old age groups, about 20 % more girls are not consuming recommended amounts compared to boys of the same age. By ages 9–18, girls are consuming approximately 0.7 servings less of dairy compared to their male counterparts.

Gender differences in dairy consumption persist into adulthood. In fact, less than 1 % of American females ages 9 and older consume the recommended number of dairy servings. Fewer than 4 % of men ages 19–50 are getting the recommended daily number of dairy servings and less than 1 % of older men. About 11 % percent of people 2 years of age and older obtain 3 servings of dairy indicating this recommendation is feasible (data not shown).

### Impact of dairy group consumption on nutrient adequacy

#### Overview

**Fig. 1:**
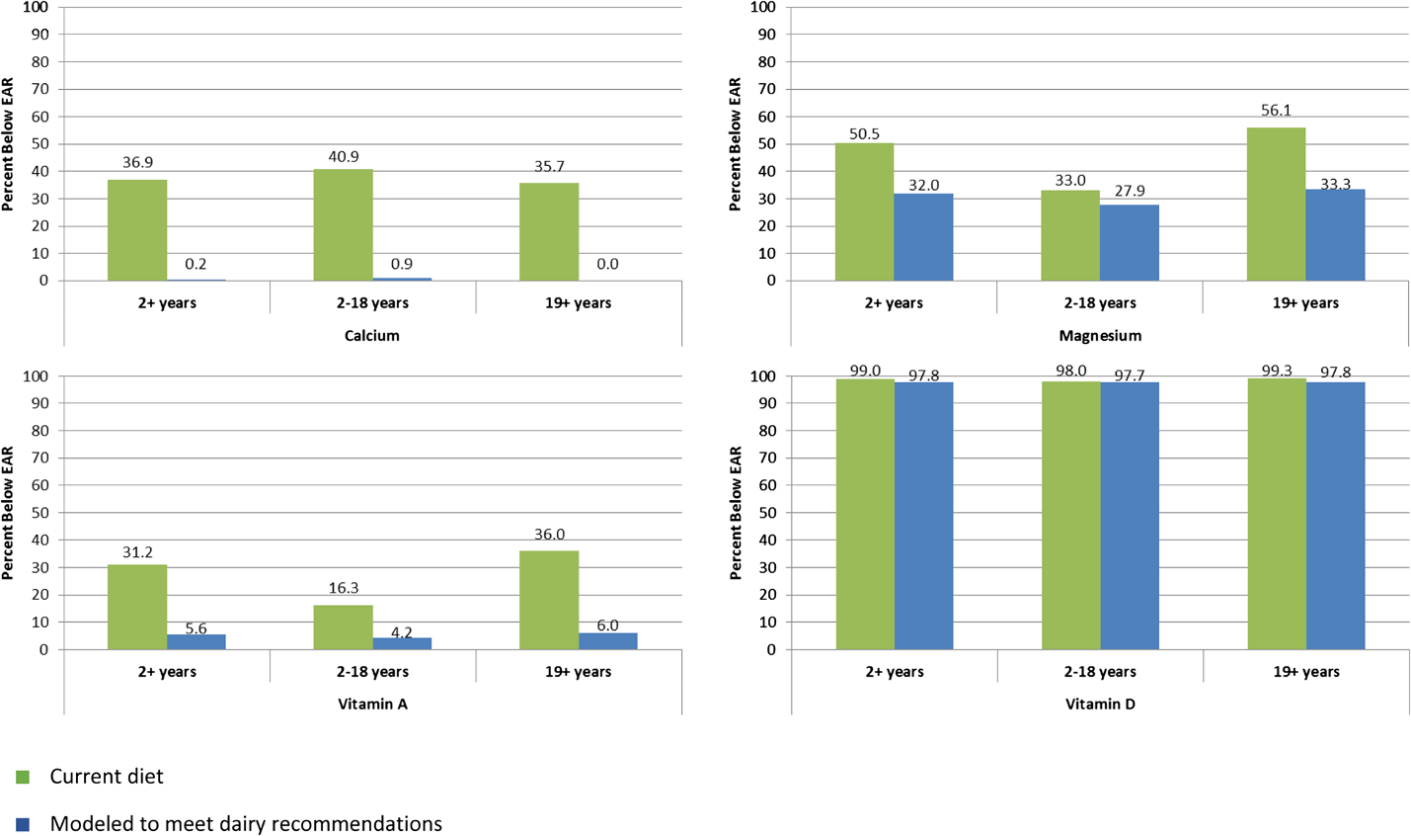
Percentage of individuals with nutrients intakes below the EAR. Calcium, magnesium, vitamin A and vitamin D intakes were determined based on current food intakes and diets modeled at recommended dairy servings. The percentage of Americans 2 years and older, children 2–18 years and adults 19 years and older who are below the EAR for each nutrient is shown. (Green: current diet, Blue: modeled to meet dairy recommendations)

Given the large gaps in the U.S. diet for calcium, magnesium, vitamin A, vitamin D and potassium, the percentage of individuals at and above the AI for potassium and below the EAR for all other nutrients was examined at current dairy intakes and with diet modeling of dairy intake increased to recommended amounts (Fig. 1, Tables 2, 3, 4, 5).

**Table 2:** Dairy and nutrient intakes at current dairy intakes and diets modeled at recommended dairy servings

		All individuals					
Age groups		2–3 years	4–8 years	9–18 years	19–50 years	51–70 years	71+ years
Total dairy, cup/equivalents	Current	2.38 ± 0.11	2.04 ± 0.05	2.05 ± 0.06	1.65 ± 0.04	1.46 ± 0.04	1.28 ± 0.03
	Modeled	2.43 ± 0.10	2.39 ± 0.04	3.00 ± 0.04	3.03 ± 0.03	2.99 ± 0.03	2.91 ± 0.02
Calcium, mg/d	Current	1037 ± 35	975 ± 17	1047 ± 21	1025 ± 15	933 ± 14	814 ± 13
	Modeled	1054 ± 34	1081 ± 15	1329 ± 16	1436 ± 12	1387 ± 11	1300 ± 11
Magnesium, mg/d	Current	200 ± 4	211 ± 2	247 ± 4	307 ± 5	302 ± 4	256 ± 4
	Modeled	201 ± 4	218 ± 2	266 ± 4	335 ± 5	332 ± 4	289 ± 4
Protein, g/d	Current	53.3 ± 1.2	59.4 ± 0.7	75.2 ± 1.2	87.2 ± 1.0	79.3 ± 1.1	64.3 ± 1.2
	Modeled	53.7 ± 1.2	62.5 ± 0.6	83.3 ± 1.1	98.9 ± 0.9	92.2 ± 1.1	78.1 ± 1.1
Vitamin A, RAE/d	Current	581 ± 15	579 ± 11	598 ± 18	599 ± 13	664 ± 12	658 ± 17
	Modeled	587 ± 15	615 ± 11	692 ± 16	736 ± 12	815 ± 11	819 ± 16
Vitamin D, μg/d	Current	7.1 ± 0.2	5.8 ± 0.1	5.3 ± 0.1	4.7 ± 0.1	4.9 ± 0.1	4.7 ± 0.1
	Modeled	7.2 ± 0.2	6.4 ± 0.1	6.7 ± 0.1	6.8 ± 0.1	7.3 ± 0.1	7.2 ± 0.1
Potassium, mg/d	Current	2035 ± 37	2049 ± 23	2261 ± 42	2705 ± 36	2799 ± 38	2457 ± 39
	Modeled	2049 ± 37	2134 ± 23	2485 ± 39	3031 ± 34	3160 ± 36	2843 ± 37
Calories, kcal/d	Current	1456 ± 23	1728 ± 16	2088 ± 24	2276 ± 22	2011 ± 20	1649 ± 27
	Modeled	1460 ± 23	1757 ± 15	2164 ± 24	2388 ± 21	2135 ± 20	1781 ± 26
Saturated Fat, g/d	Current	19.2 ± 0.6	22.3 ± 0.4	26.6 ± 0.4	28.0 ± 0.4	25.5 ± 0.4	20.2 ± 0.4
	Modeled	19.3 ± 0.6	22.7 ± 0.4	27.5 ± 0.4	29.1 ± 0.4	26.9 ± 0.4	21.7 ± 0.4
Sodium, mg/d	Current	2113 ± 44	2617 ± 37	3392 ± 70	3742 ± 39	3375 ± 46	2717 ± 52
	Modeled	2124 ± 44	2681 ± 37	3563 ± 69	3992 ± 37	3651 ± 45	3013 ± 51

**Table 3:** Percentage of individuals with nutrient intakes below the EAR at current food intakes and diets modeled at recommended dairy servings

	Vitamin A			Vitamin D			Calcium			Magnesium		
	All	Below Recommended Dairy	Modeled at Recommended Dairy	All	Below Recommended Dairy	Modeled at Recommended Dairy	All	Below Recommended Dairy	Modeled at Recommended Dairy	All	Below Recommended Dairy	Modeled at Recommended Dairy
	% ± SE	% ± SE	% ± SE	% ± SE	% ± SE	% ± SE	% ± SE	% ± SE	% ± SE	% ± SE	% ± SE	% ± SE
Males												
2–3 years	0.0 ± 0.0	0.0 ± 0.0	0.0 ± 0.0	91.8 ± 1.9	100.0 ± 0.0	91.7 ± 1.9^a^	0.4 ± 0.4	3.0 ± 3.0	0.0 ± 0.0	0.3 ± 0.3	2.5 ± 2.5	0.0 ± 0.0
4–8 years	0.0 ± 0.0	0.0 ± 0.0	0.0 ± 0.0	97.9 ± 0.4	99.4 ± 0.3	97.8 ± 0.4^a^	6.1 ± 1.0	10.5 ± 1.7	0.0 ± 0.0^a^	0.0 ± 0.0	0.0 ± 0.0	0.0 ± 0.0
9–18 years	25.8 ± 1.4	32.6 ± 1.5	13.0 ± 1.3^a^	96.8 ± 0.8	99.8 ± 0.1	96.0 ± 0.7^a^	40.8 ± 2.5	52.1 ± 2.6	1.0 ± 0.3^a^	47.6 ± 1.6	49.9 ± 2.1	43.0 ± 1.7
19–50 years	48.0 ± 1.2	49.8 ± 1.2	15.1 ± 0.9^a^	99.0 ± 0.3	99.5 ± 0.3	96.8 ± 0.5^a^	6.9 ± 0.6	7.2 ± 0.7	0.0 ± 0.0^a^	51.7 ± 1.6	53.2 ± 1.5	34.2 ± 1.5^a^
51–70 years	34.7 ± 1.6	35.4 ± 1.6	5.2 ± 0.6^a^	98.0 ± 0.4	98.6 ± 0.4	96.1 ± 0.5^a^	12.4 ± 1.1	12.6 ± 1.1	0.0 ± 0.0^a^	59.6 ± 1.5	60.7 ± 1.5	40.5 ± 1.9^a^
71+ years	23.4 ± 1.5	23.6 ± 1.5	1.7 ± 0.5^a^	98.7 ± 0.4	99.1 ± 0.3	96.1 ± 0.6^a^	72.0 ± 1.7	72.7 ± 1.7	0.0 ± 0.0^a^	88.5 ± 1.4	88.9 ± 1.4	75.0 ± 2.2^a^
Females												
2–3 years	0.1 ± 0.1	0.6 ± 0.6	0.0 ± 0.0	95.8 ± 1.0	100.0 ± 0.0	95.8 ± 1.0^a^	0.0 ± 0.0	0.0 ± 0.0	0.0 ± 0.0	0.0 ± 0.0	0.0 ± 0.0	0.0 ± 0.0
4–8 years	0.0 ± 0.0	0.0 ± 0.0	0.0 ± 0.0	99.9 ± 0.0	100.0 ± 0.0	99.9 ± 0.1	17.7 ± 1.8	22.9 ± 2.1	0.0 ± 0.0^a^	0.0 ± 0.0	0.0 ± 0.0	0.0 ± 0.0
9–18 years	29.3 ± 2.0	29.6 ± 2.0	1.7 ± 0.4^a^	99.9 ± 0.1	99.9 ± 0.1	99.8 ± 0.1	85.3 ± 1.2	86.0 ± 1.3	2.0 ± 0.5^a^	63.8 ± 1.6	64.4 ± 1.7	51.4 ± 1.8^a^
19–50 years	40.3 ± 1.5	40.5 ± 1.5	2.0 ± 0.3^a^	99.9 ± 0.1	99.9 ± 0.0	99.5 ± 0.2	34.5 ± 1.3	34.5 ± 1.3	0.0 ± 0.0^a^	55.8 ± 1.9	56.0 ± 1.9	28.8 ± 1.5^a^
51–70 years	17.8 ± 1.7	17.8 ± 1.7	0.4 ± 0.1^a^	99.8 ± 0.1	99.8 ± 0.1	98.4 ± 0.4^a^	81.7 ± 1.8	81.7 ± 1.8	0.0 ± 0.0^a^	44.7 ± 1.9	44.7 ± 1.9	17.3 ± 1.0^a^
71+ years	15.3 ± 1.0	15.3 ± 1.0	0.1 ± 0.1^a^	99.9 ± 0.1	99.9 ± 0.1	98.6 ± 0.4^a^	91.1 ± 0.6	91.1 ± 0.6	0.0 ± 0.0^a^	75.8 ± 1.8	75.8 ± 1.8	44.8 ± 1.6^a^

^a^95 % Confidence intervals of values from those below recommended and modeled intake do not overlap

**Table 4:** Percentage of individuals with nutrient intakes below the EAR at current food and dietary supplement intakes and diets modeled at recommended dairy servings

	Calcium		Magnesium		Vitamin A		Vitamin D	
	NHANES 2007–2010	Modeled at Recommended Dairy	NHANES 2007–2010	Modeled at Recommended Dairy	NHANES 2007–2010	Modeled at Recommended Dairy	NHANES 2007–2010	Modeled at Recommended Dairy
	% ± SE	% ± SE	% ± SE	% ± SE	% ± SE	% ± SE	% ± SE	% ± SE
Males								
2–3 years	0.4 ± 0.4	0.0 ± 0.0	0.3 ± 0.3	0.0 ± 0.0	0.0 ± 0.0	0.0 ± 0.0	63.5 ± 2.7	63.0 ± 2.7
4–8 years	5.8 ± 1.1	0.0 ± 0.0^a^	0.0 ± 0.0	0.0 ± 0.0	0.0 ± 0.0	0.0 ± 0.0	66.7 ± 2.9	64.5 ± 2.8
9–18 years	38.6 ± 2.6	1.0 ± 0.3^a^	45.2 ± 1.6	40.8 ± 1.6	22.8 ± 1.3	11.5 ± 1.3^a^	84.8 ± 1.6	82.6 ± 1.5^a^
19–50 years	6.2 ± 0.6	0.0 ± 0.0^a^	44.9 ± 1.6	29.0 ± 1.4^a^	40.0 ± 1.4	12.5 ± 0.8^a^	81.6 ± 1.0	77.1 ± 1.2^a^
51–70 years	9.2 ± 1.0	0.0 ± 0.0^a^	44.6 ± 1.8	28.4 ± 2.0^a^	25.7 ± 1.6	4.1 ± 0.6^a^	62.9 ± 1.8	59.2 ± 1.9
71+ years	49.6 ± 2.1	0.0 ± 0.0^a^	66.4 ± 2.4	52.3 ± 2.0^a^	15.1 ± 1.7	12.5 ± 0.8^a^	54.9 ± 2.4	51.9 ± 2.5
Females								
2–3 years	0.0 ± 0.0	0.0 ± 0.0	0.0 ± 0.0	0.0 ± 0.0	0.1 ± 0.1	0.0 ± 0.0	72.6 ± 2.4	72.5 ± 2.4
4–8 years	17.1 ± 1.8	0.0 ± 0.0^a^	0.0 ± 0.0	0.0 ± 0.0	0.0 ± 0.0	0.0 ± 0.0	79.3 ± 2.2	75.2 ± 2.7
9–18 years	81.9 ± 1.5	2.0 ± 0.5^a^	61.2 ± 1.7	48.3 ± 1.8^a^	25.5 ± 1.7	1.7 ± 0.4^a^	89.4 ± 1.4	87.3 ± 1.6
19–50 years	26.2 ± 1.0	0.0 ± 0.0^a^	47.3 ± 1.9	24.5 ± 1.4^a^	31.5 ± 1.4	2.0 ± 0.3^a^	76.0 ± 1.2	72.3 ± 1.2
51–70 years	44.0 ± 4.0	0.0 ± 0.0^a^	30.9 ± 1.6	11.5 ± 0.9^a^	12.7 ± 1.2	0.4 ± 0.1^a^	51.5 ± 1.7	47.6 ± 1.8
71+ years	51.4 ± 2.1	0.0 ± 0.0^a^	54.2 ± 1.7	28.4 ± 1.5^a^	9.8 ± 0.8	0.0 ± 0.0^a^	52.4 ± 2.0	48.2 ± 2.2

^a^95 % Confidence intervals of values from those below recommended and modeled intake do not overlap

**Table 5:** Percentage of individuals with potassium intakes above the AI from foods alone or foods plus dietary supplements and diets modeled at recommended dairy servings

	Potassium (Food alone)		Potassium (Food + Supplements)	
	NHANES 2007–2010	Modeled at Recommended Dairy	NHANES 2007–2010	Modeled at Recommended Dairy
	% ± SE	% ± SE	% ± SE	% ± SE
Males				
2–3 years	1.8 ± 1.0	1.8 ± 1.0	1.8 ± 1.0	1.8 ± 1.0
4–8 years	0.0 ± 0.0	0.0 ± 0.0	0.0 ± 0.0	0.0 ± 0.0
9–18 years	0.2 ± 0.1	0.2 ± 0.1	0.2 ± 0.1	0.2 ± 0.1
19–50 years	1.3 ± 0.3	2.0 ± 0.3^a^	1.6 ± 0.3	2.3 ± 0.4
51–70 years	0.5 ± 0.3	1.3 ± 0.4^a^	0.6 ± 0.3	1.7 ± 0.5^a^
71+ years	0.1 ± 0.1	0.1 ± 0.1	0.1 ± 0.1	0.1 ± 0.1
Females				
2-3 years	0.3 ± 0.2	0.3 ± 0.2	0.3 ± 0.2	0.3 ± 0.2
4-8 years	0.0 ± 0.0	0.0 ± 0.0	0.0 ± 0.0	0.0 ± 0.0
9-18 years	0.1 ± 0.1	0.1 ± 0.1	0.1 ± 0.1	0.1 ± 0.1
19-50 years	0.1 ± 0.1	0.1 ± 0.1	0.1 ± 0.1	0.1 ± 0.1
51-70 years	0.0 ± 0.0	0.0 ± 0.0	0.0 ± 0.0	0.1±0.1
71+ years	0.0 ± 0.0	0.0 ± 0.0	0.0 ± 0.0	0.0 ± 0.0

^a^95 % Confidence intervals of values from those below recommended and modeled intake do not overlap

In general, a large percentage (30–99 %) of individuals over the age of 2 years in the U.S. fall below the EAR for calcium, magnesium, vitamin A and vitamin D (Fig. 1). When modeling dairy intake at recommended amounts, essentially all Americans would meet recommended calcium intakes. The percentage of the population below the EAR for vitamin A would be significantly improved, with only 4.2 % of children and 6.0 % of adults falling below the EAR if dairy recommendations were met. If dairy intake was increased, magnesium intakes would also significantly improve, however a large portion of Americans would still fall below the recommended intakes of magnesium with 27.9 % of children and one–third of adults below the EAR. Even with adequate dairy intake, vitamin D intakes would still be far below the new 2010 EAR for vitamin D.

#### Calcium

Diet modeling with the recommended number of dairy servings virtually eliminated inadequate calcium intakes across all age and gender categories (Table 3). Females (ages 4–71+ years) have a much higher prevalence of inadequate calcium intake and would benefit the most from increasing dairy intake.

In general, the prevalence of inadequate calcium intake was lower for calcium intake from food and supplements (Table 4) compared to that from food alone (Table 3). In both cases, modeling the recommended amounts of dairy product consumption would reduce the prevalence of inadequate intake to effectively zero.

#### Magnesium

Adequate dairy intake would improve magnesium intakes in teens and adults, especially older adults, in both genders (Table 3). Females ages 9–71+ years would benefit more from consuming the recommended amounts of dairy even though the prevalence of inadequate magnesium intake was often higher among women. Even with adequate dairy intake, inadequate magnesium intake would remain high among males ages 9–71+ at 34–75 % prevalence and among women ages 9–71+ at 17–51 %.

In general, the prevalence of inadequate magnesium intake was lower when examining magnesium intake from food and supplements (Table 4) compared to that from food alone (Table 3). In both cases, modeling the recommended amounts of dairy intake would significantly reduce the prevalence of inadequate magnesium intake for males 19 years and older and females 9 years and older.

#### Vitamin A

Vitamin A intake would be greatly improved by adequate dairy intake especially among older adults and women (Table 3). Adult men (ages 19–71+) had a higher absolute prevalence of inadequate vitamin A intake than adult women (7.7 %, 16.9 %, and 8.1 % higher prevalence for ages 19–50, 51–70, and 71+, respectively), and the modeled increase in dairy intake to recommended amounts largely restored vitamin A intakes to adequate intakes for men ages 71+ and women ages 9–71+. For males ages 9–70 years, inadequate vitamin A intakes were only partially ameliorated (5.2–15.1 % prevalence remained) when modeled at recommended amounts of dairy intake.

In general, the prevalence of inadequate vitamin A intake was lower for vitamin A intake from food and supplements (Table 3) compared to that from food alone (Table 3). In both cases, modeling the recommended amounts of dairy product consumption would significantly reduce the prevalence of inadequate vitamin A intake for both males and females 9 years of age and older.

#### Vitamin D

In contrast to the above, modeling adequate dairy intake did not reduce the prevalence of inadequate vitamin D intakes because intakes are far from recommended for a large portion of the population. The greatest improvement would be among males ages 71+ with a reduction in absolute prevalence of 2.6 %. Smaller reductions among other age-gender groups would leave the prevalence of inadequate vitamin D intake essentially unchanged at 91–99 % depending on age-gender group.

#### Potassium

Potassium data are expressed as percent of the U.S. population consuming at or above the AI established for each age-gender group (Table 5). Less than 2 % of Americans in any age-gender group consume at or above the AI for potassium. Modeling recommended amounts of dairy products slightly increased the proportion of men 19–70 years old with potassium intakes above the AI from foods alone and men 51–70 years old from foods and supplements, however the total at or above the AI remained below 2 % for all age-gender groups, indicating that the majority of Americans would still not meet potassium intake recommendations.

#### Other vitamins and minerals

This modeling study also examined the impact of recommended amounts of dairy consumption on adequacy of intakes of riboflavin, vitamin B12, phosphorus and zinc (data not shown). For riboflavin and vitamin B12, the prevalence of inadequate intakes remained low regardless of dairy intake, i.e. < 5 % prevalence for all age-gender groups.

For 9–18 year old girls, 31.5 ± 2.0 % (food alone) or 30.8 ± 2.0 % (food and supplements) had intakes of phosphorus below the EAR based on their current diet; however, that shortfall was completely resolved when recommended amounts of dairy were modeled into their diets. For 9–18 year old boys, the prevalence of inadequate phosphorus intake was 3.2 ± 0.7 % (food alone) or 3.1 ± 0.7 % (food and supplements) and that shortfall was also completely resolved with recommended amounts of dairy in the model. Inadequate intake of phosphorus did not exceed 0.1 % prevalence in any other age group regardless of dairy intake.

There was a low prevalence of inadequate zinc intake from food alone or food and supplements for men ages 71+ (6.6 ± 1.2 % and 5.3 ± 0.9 %, respectively) and girls ages 4–8 (7.2 ± 1.1 % and 6.7 ± 1.1 %, respectively). Modeling recommended amounts of dairy reduced the prevalence in both age-gender groups to less than 0.1 %. Inadequate zinc intake did not exceed 3 % for any other age-gender group.

#### Calories, fat and sodium

Modeling adequate numbers of dairy servings into the U.S. population’s current diet would modestly increase calories, saturated fat and sodium intakes (Table 2). The largest increase in dairy servings would be in the ages 71+ group (both genders) where an additional 1.6 servings of dairy products per day would lead to an 8 % increase in calories, 7 % increase in saturated fat and 11 % increase in sodium intake. The other age and gender groups require a smaller increase in dairy servings, and therefore there would be a smaller increase in calories, fat and sodium.

## Discussion

A majority of children over the age of three and nearly all adults in the U.S. do not consume the daily recommended number of dairy servings (3 servings for individuals 9 years and older, 2.5 servings for children 4–8 years, and 2 servings for children 2–3 years). This analysis demonstrates that if Americans consumed the recommended amounts of dairy products, it would provide sufficient calcium for all people to meet the EAR. Consuming the recommended amounts of dairy also would provide sufficient vitamin A for almost all women and greatly reduce the prevalence of inadequate vitamin A intake among men, and inadequate magnesium intake among most Americans. But, consuming recommended amounts of dairy would not make a meaningful impact on the prevalence of inadequate vitamin D and potassium intakes for most age-gender groups. That said, intake of these nutrients increased substantially with higher dairy intake (26–53 % and 10–16 %, respectively for those 9 years of age and older). So a combination of greater dairy intake and increased consumption of other vitamin D and potassium-containing foods is likely needed to improve intakes, while meeting overall calorie goals. This analysis has updated and expanded our understanding of how achieving recommended amounts of dairy could help the U.S. population better meet nutritional guidelines, especially those for certain nutrients of concern.

This analysis (based on 2007–2010 NHANES data) reports a higher prevalence of not consuming the recommended number of daily dairy servings compared to Krebs-Smith, et al. [5] which used the 2001–2004 NHANES data. This was the case for young children, as well as girls 9–18 years old. In fact, 99 % of females 9 years and older currently do not meet dairy recommendations. If this is not addressed, this six year trend of fewer women and children consuming adequate amounts of dairy foods could lead to even greater nutritional gaps in the future.

Low calcium intake is prevalent enough in the U.S. to be of public health concern [1] and coupled with below recommended dairy consumption there is the potential to impact skeletal growth in early life and bone health later in life, including bone mineral density and bone metabolism [9, 25–27]. In keeping with DGA goals for a food-based approach to a nutritionally adequate U.S. diet [1], this analysis indicates that consuming recommended amounts of dairy by itself has the potential to reduce inadequate calcium intake in all age-gender groups (Table 3). Dietary supplements play a role in helping some population groups get more calcium, with the largest impact in older men (71+ years) and women (51+ years), however about half of these adults are still consuming below the EAR (Table 4).

In our modeling adequate dairy intake partially offset low magnesium intakes among U.S. adults by increasing magnesium consumption by 8–13 % in those 9 years of age and older (Table 4), but it still left up to 52.3 % of men and 48.3 % of females falling below adequate magnesium intake. Ensuring adequate magnesium intake is important for bone remodeling and energy metabolism and therefore additional considerations are warranted to further close this gap. An additional serving of dairy may be one approach to help Americans get more magnesium since increasing dairy from current to recommended amounts reduced the percentage of people below the EAR by as much as 26 %. Based on current dietary intakes, milk is the primary contributor of magnesium to the diet of children [3] and the second highest contributor for adults [4] in the U.S. compared to other foods. Other magnesium-rich foods such as nuts and beans should also be considered to help fully close this nutrient gap (1).

While vitamin A deficiency is not a widespread concern in the U.S., a significant portion of older children and adults have inadequate intake of this vitamin. Vitamin A plays a key role in immune function, eye and skin health, and dietary practices to improve intake for these population groups should be examined. Notably, adequate dairy intake would largely resolve inadequate vitamin A intake among women while leaving a much-reduced prevalence of inadequate vitamin A intake of 4–13 % in males ages 9–71+. This is expected given the higher prevalence of inadequate vitamin A intake among adult males and the smaller increase in dairy servings needed to meet dairy group recommendations among males ages 9–18 and ages 71 and older compared to women of the same age. Dietary interventions to ensure males consume sufficient vitamin A should therefore be explored. This could include recommending four servings of low-fat dairy foods for males, particularly milk as NHANES consumption data indicates it is also the leading food source of vitamin A for children [3] and adults [4] in the U.S, as well as increasing intake of orange and dark green vegetables which are rich sources of vitamin A.

Vitamin D is a key nutrient for bone health and research indicates it may play a role in a number of other health outcomes as well, although more research is needed [9]. Potassium is an important nutrient in blood pressure management. Meeting dairy recommendations had less impact on vitamin D and potassium adequacy of the diet. While milk contributes the most of these two nutrients to the U.S. diet compared to other foods and beverages [3, 4], the data indicate that increasing a single food group will not be sufficient to meet recommended vitamin D and potassium intake based on current diet patterns, and additional dietary strategies would be needed. As has been noted by the Institute of Medicine [9], the most appropriate measure of vitamin D adequacy is serum 25-hydroxyvitamin D to take into account the contributions from both sunlight and diet. Based on this measure, most of the U.S. population obtains adequate amounts of vitamin D and therefore both diet and environmental factors should to be evaluated when determining vitamin D needs. This analysis also indicates that in cases where a person limits their exposure to sunlight to the point of deficiency or is otherwise at risk due to dark skin or advanced age, it may not be possible to meet vitamin D requirements without also consuming foods like canned fish with bones [1] or taking vitamin D supplements. Similarly for potassium, based on current diet patterns, one single dietary behavior change is unlikely to ensure sufficient potassium intake and thus a multi-faceted approach that includes increased consumption of multiple potassium-rich foods is needed to significantly shift population intake.

Because adding additional foods to the current U.S. diet (in this modeling study, the USDA dairy composite serving) would provide additional calories, saturated fat, and sodium, it is important to determine what other dietary changes would be needed to meet overall nutrition goals. Increasing the number of servings of dairy per day to recommended amounts had the largest impact on the diet composition of age-gender groups typically consuming the lowest amounts of dairy (Table 2), i.e. older adults of both genders. In the group with the largest increase in dairy servings, adults ages 71 years and older, the additional 1.6 servings a day of the dairy composite increased calories by 8 %, saturated fat by 7 %, and sodium by 11 %. At the same time, this modeled diet eliminated inadequate calcium intake and greatly improved vitamin A and magnesium intakes. Persons looking to lower calories and limit certain nutrients can choose lower fat, lower sugar and/or lower sodium choices within the Dairy Group or reduce intake of other foods such as desserts and refined grains [28]. Future work should also model the replacement of less nutrient dense foods (e.g., certain snacks and sweetened beverages) with dairy foods, and examine the impact of adding dairy servings with a composition like those typically consumed rather than using the USDA ideal composite.

Taken together, these analyses indicate that consumption of dairy products below recommended levels is contributing to inadequate nutrient intakes for some nutrients, particularly for adolescents, older adults and females. There is a need to determine the most effective dietary behaviors that prevent the decline in dairy intake that occurs in early childhood, particularly since research indicates that food preferences and behaviors develop during the first five years of life [29, 30]. A recent report from the U.S. Department of Agriculture indicates under-consumption of milk is due to decreased frequency of milk intake, particularly at mid-day and evening meals, and therefore recommends encouraging drinking milk at meals as a key strategy to ensure nutritional adequacy [31]. This study focused on the population impact of increasing dairy intake and therefore used the EAR and AI to assess nutrient adequacy; however, on an individual nutritional counseling basis the RDA should be used and some nutrient gaps may remain that require either additional servings of dairy or other nutritional interventions to ensure adequate nutrient intake.

Strengths of this study are the use of a large, nationally representative database to examine food and nutrient intakes and using advanced statistical techniques to assess usual intake in numerous age/gender groups.

Limitations of this study include use of self-reported intakes which on an individual basis may over- or under-represent actual intake. Additionally, we modeled increased dairy intake with a USDA composite of dairy consumption, which contains a greater proportion of lower fat dairy options than currently consumed.

## Conclusions

Americans’ diets are far from ideal and as a result the intake of several essential nutrients is below recommended intakes. Increasing dairy group consumption, including milk, cheese and yogurt, to the DGA recommended amounts is one practical dietary change that could significantly improve the population’s adequacy for certain vitamins and minerals that are currently under-consumed. In particular, meeting dairy recommendations would result in virtually all Americans meeting the EAR for calcium, most population groups meeting the EAR for vitamin A and significantly fewer adolescent girls and adults falling below the EAR for magnesium. Including four servings of dairy in dietary patterns may be an approach to close the remaining vitamin A gaps for adolescent boys and young men and the magnesium gaps for adolescent girls and adults. While increasing dairy intake would minimally improve the intakes of vitamin D and potassium for some age-gender groups, there would still be a large portion of Americans with inadequate intakes of these nutrients and therefore additional interventions should be considered. Diets that include higher dairy intake also may help support public health goals. As indicated in the 2010 DGA, intake of dairy products is associated with improved bone health, especially in children and adolescents, and with a reduced risk of cardiovascular disease and type 2 diabetes and with lower blood pressure in adults.

## Abbreviations

NHANES: National Health and Nutrition Examination Survey
DRI: Dietary Reference Intakes
USDA: United States Department of Agriculture
WWEIA: What We Eat in America
EAR: Estimated Average Requirement
DGA: Dietary Guidelines for Americans
AI: Adequate Intake
RAE: Retinol Activity Equivalents
RDA: Recommended Dietary Allowance
